# Efficient Adsorption and Utilisation of Methylene Blue by NaOH-Modified Nanocellulose–Polyacrylamide Interpenetrating Network Gels

**DOI:** 10.3390/gels11040252

**Published:** 2025-03-27

**Authors:** Yanan Wang, Yanan Lu, Hao Zhong, Minghui Guo, Jingkui Li

**Affiliations:** 1 Key Laboratory of Bio-Based Material Science and Technology of Ministry of Education, Material Science and Engineering College, Northeast Forestry University, Harbin 150040, China; ynwangnefu@163.com (Y.W.); zhong19990901@126.com (H.Z.); 2 Inner Mongolia Academy of Agricultural and Animal Husbandry Sciences, Hohhot 010000, China; ynlunefu@163.com; 3 College of Science, Northeast Forestry University, Harbin 150040, China

**Keywords:** NaOH-modified, nanocellulose, polyacrylamide, MB-releasing, gel

## Abstract

To solve the problem of dye contamination caused by methylene blue (MB), a one-step synthesised nanocellulose (CNF) and polyacrylamide (PAM) gel network was modified by using NaOH in this study, and the prepared samples were analysed for their micromorphology, chemical structure, and adsorption-release properties. The findings demonstrated that the maximum adsorption capacity of the CNF-PAM5% was 172.08 mg/g, which followed the quasi-second-order kinetic model and the Freundlich adsorption model. The adsorption of the gel increased with the increase of the NaOH-modified concentration. However, the adsorption efficiency of the CNF-PAM5% could still reach 85% after four cycles, and the CNF-PAM5% remained intact without signs of fragmentation after 4 h of stirring and water impact, which was attributed to the introduction of CNF into the PAM network to effectively improve the mechanical properties of the gel. Moreover, toxicity tests showed no significant difference in the amount of cellular activity, even when the volume of the CNF-PAM5% sample was increased up to 10-fold. This gel, which exhibits low toxicity and excellent recycling properties, serves to reduce environmental impact during the adsorption process. Furthermore, the potential exists for utilising the gel’s methylene blue-releasing (MB) properties as a fungicide for fish.

## 1. Introduction

The development of the textile industry has exacerbated the problem of water pollution [1], in which the undesirable visual impact and toxic effects upon decomposition, which may contaminate soils and surface waters, make them one of the major global sources of environmental pollution [2,3,4]. In addition to this, it also accumulates and causes irreversible damage in the human body through, for example, the water cycle and the use of natural resources [5,6]. Therefore, there is an urgent need to develop adsorbents for purifying dyes from water resources [7]. Wastewater treatment primarily employs several key methods, including filtration through membranes [8], biodegradation processes [9], adsorption techniques, and photocatalytic reactions [10]. Among these methods, gel adsorbents, characterised by their excellent adsorption properties, can rapidly and efficiently adsorb large quantities of harmful substances in wastewater and are consequently widely used in the wastewater treatment industry [11,12,13,14,15]. To minimise the secondary pollution of adsorbent materials to aquatic environments, numerous natural polymers have emerged as a research focus in the development of adsorbent materials in recent years [16]. Among these, cellulose, as the world’s most abundant renewable natural biopolymer, is ubiquitous in various organisms and possesses a high density of hydroxyl groups [17]. Through a series of chemical modifications, its properties can be significantly enhanced, thereby enabling the multifaceted realisation of the anticipated potential applications of cellulose-based materials [18]. Moreover, cellulose can be processed to obtain high-performance nano-fibrillated cellulose (CNF). In comparison with cellulose, nanocellulose has been shown to possess a higher aspect ratio, an enhanced compatibility for hybridisation with other materials, improved processability, and the potential to form a gel–fibre network [19]. The porous network structure of composite gels prepared using CNF provides a substantial number of adsorption sites. Additionally, the abundant hydroxyl groups facilitate the facile functionalisation of the cellulose gels, enabling the preparation of functional separation materials tailored for various pollutants of significant interest [20,21,22,23]. Among them, modification using an alkali treatment can enhance the adsorption performance of gels in wastewater treatment [24,25]. On the one hand, alkali treatments can optimise the microstructure of the material or change the surface charge to enhance the physical adsorption capacity; on the other hand, they can introduce or improve functional groups on the gel surface, providing more chemisorption active sites [26]. In addition, alkali treatments can strengthen the stability of the gel network to maintain efficient adsorption in complex water quality, and this technique provides some new ideas for the development of green and efficient multifunctional water treatment materials [27].

Methylene blue (MB) is a cationic thiazine dye with an alkaline aqueous solution that exhibits toxicity. It finds extensive applications in industries such as medicine and aquaculture, among others [28]. A commonly used wastewater dye, methylene blue imposes a significant burden on aquatic environments. Therefore, the development of recyclable and cost-effective adsorbents that can efficiently remove methylene blue is imperative [29]. Despite the significant hazards associated with methylene blue in dyeing wastewater, it plays an important role in aquaculture, particularly as a preservative and disinfectant in ornamental fish farming [30]. It effectively prevents water mould, white spot disease, and tail rot in fish, and is also employed for quarantining newly acquired fish, making it a commonly used medication in fish culture [31]. Typically, it is challenging to precisely control the dosage when adding MB directly. However, by placing the adsorbed gel into the fish tank and allowing it to release MB gradually, one can effectively monitor the health status of the fish while ensuring better control over both the rate and quality of the MB release.

In this study, we incorporated CNF extracted from wood into the base polyacrylamide (PAM) gel system. Subsequently, we modified the CNF-PAM composite using sodium hydroxide (NaOH) solution. This approach aimed to develop adsorbent materials targeting methylene blue (MB) through a simple and cost-effective synthesis method. The effect of the NaOH modification on the chemical structure and properties of the CNF-PAM gel adsorbent was explored through characterisation and testing, and the mechanism of adsorption was investigated using kinetic modelling. Furthermore, the release characteristics of the completed adsorbed gel were evaluated to investigate its potential as a controlled-release fungicide for fish, thereby enhancing the utilisation efficiency of the gel agent and minimising the environmental impact.

## 2. Results and Discussion

### 2.1. Characterisation of Gels

**Figure 1:**
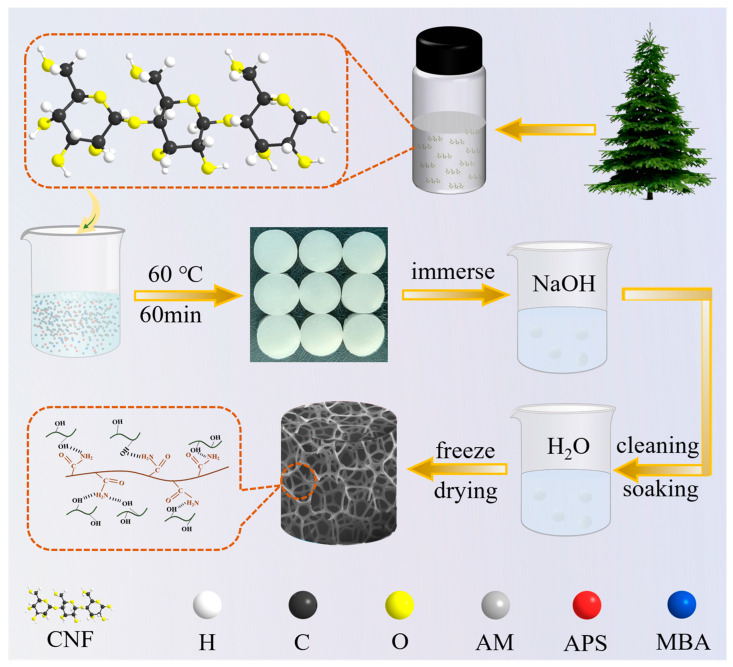
Schematic flow chart for the preparation of gel.

**Figure 2:**
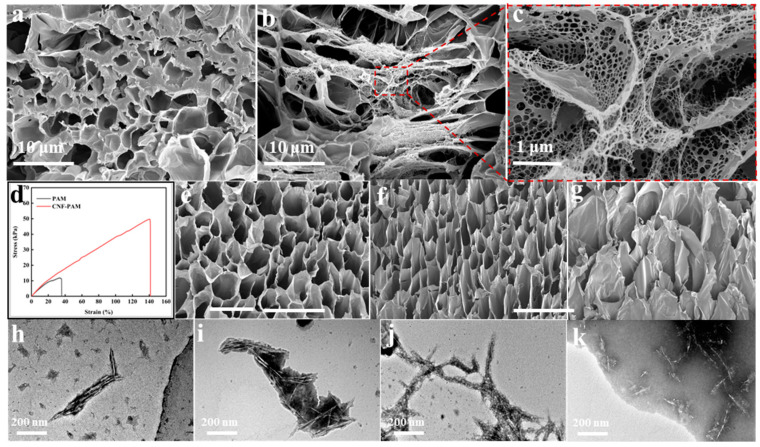
Scanning electron micrographs (SEM) of PAM (**a**); CNF-PAM (**b**); CNF-PAM localised magnification (**c**); stress–strain diagram of gels (**d**); CNF-PAM1% (**e**); CNF-PAM5% (**f**); and CNF-PAM10% (**g**). Transmission electron micrographs (TEM) of CNF (**h**). CNF was subjected to treatment with varying concentrations of NaOH, specifically 1%, 5%, and 10% (**i**–**k**).

Nanocellulose (CNF) derived from wood was incorporated into the PAM network, followed by modification of the gel using varying concentrations of the NaOH solution, as illustrated in Figure 1. Scanning electron microscopy (SEM) revealed that when PAM was crosslinked independently to form a gel, the resulting structure primarily consisted of large, irregularly shaped smooth pores (Figure 2a). However, upon the addition of CNF, it is evident that CNF is embedded within the pore walls of PAM, resulting in a complex dual-network gel structure. Upon magnification of a portion of this structure, it becomes apparent that the mesh in the spider web-like gel structure is uniformly dense, with CNF serving as a reinforcing agent for the PAM gel network, thereby enhancing the mechanical strength of the CNF-PAM composite (Figure 2b,c). To evaluate the impact of CNF on the mechanical properties of the gels, stress–strain curves for both PAM and CNF-PAM were analysed, as illustrated in Figure 2d. The introduction of CNF resulted in the enhanced rigidity and altered plastic deformation characteristics of the CNF-PAM. Specifically, the breaking strength and strain of the CNF-PAM were significantly higher than those of PAM, indicating that the incorporation of CNF effectively improved both the toughness and strength of the gels. After modification with NaOH, it was found that the network structure of the gel was gradually loosened, the pore size gradually became larger, and the diameter of the nano-fibrillated cellulose filaments on the surface of the gel gradually decreased (Figure 2e–g); when the concentration reached 10%, the network structure of the gel collapsed, and the mechanical strength was significantly reduced. To investigate the reasons for these changes, we subjected nanocellulose to NaOH treatments at concentrations of 1%, 5%, and 10%. Transmission electron microscopy (TEM) observations revealed that, in an alkaline environment, nanocellulose undergoes wetting and swelling, resulting in weakened intermolecular chain bonding and reduced cellulose diameters (Figure 2i–k). Simultaneously, PAM also exhibits swelling after the NaOH treatment, leading to weakened bonding between the CNF fibrils and the PAM network, thereby decreasing the mechanical strength of the gel.

**Figure 3:**
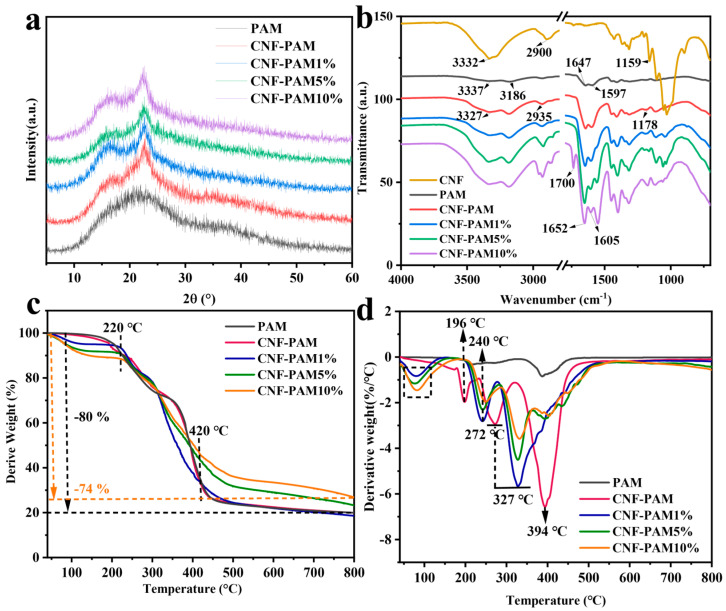
X-ray diffraction (XRD) pattern (**a**); Fourier transform infrared (FTIR) spectrum (**b**); thermogravimetric analysis (TGA) curve (**c**); and derivative thermogravimetric (DTG) curve (**d**) of the gel.

It can be observed from the XRD plots that, after the addition of CNF to the PAM system, the typical characteristic peaks of cellulose appeared at 2θ = 15.5° and 22.6°, and the degree of crystallinity was calculated to be 54.4% according to the crystallinity formula (Figure 3a) [32,33]. After modification of the CNF-PAM using NaOH, the changes in crystallinity were 47.97%, 47.6%, and 44.68% as the NaOH concentration was varied from 1% to 10%, respectively. This indicates that increasing the NaOH concentration progressively reduces the crystallinity of CNF. The cellulose crystal structure remains predominantly in type I, with no significant transition to type II observed in the XRD patterns. Treatment with NaOH disrupts the hydrogen bonds within cellulose and causes swelling of the CNF crystals, allowing sodium ions to penetrate the crystalline regions, thereby reducing crystallinity [34].

The FTIR spectra are presented in Figure 3b. In the PAM curves, the peaks at 3337 cm^−1^ and 3186 cm^−1^ can be attributed to N–H stretching vibrations, while the peak at 1647 cm^−1^ corresponds to the C=O stretching vibrations of the amide groups (amide I band). Additionally, the peak at 1597 cm^−1^ is associated with N-H bending vibrations [35,36]. The characteristic peaks at 3332 cm^−1^, 2900 cm^−1^, and 1159 cm^−1^ are assigned to the O–H stretching vibration, the C–H stretching vibration, and the C–O–C stretching vibration in CNF, respectively [37]. Compared to PAM, the N–H stretching vibrational absorption peak of the CNF-PAM shifted from 3337 cm^−1^ to 3327 cm^−1^, and the characteristic peak exhibited a notable broadening. The characteristic peaks at 2900 cm^−1^ and 1159 cm^−1^ were observed to shift to 2935 cm^−1^ and 1178 cm^−1^, respectively, compared to CNF. This shift can be attributed to the formation of intermolecular hydrogen bonds between the PAM chains and CNF [38], as well as the presence of intermolecular and intramolecular interactions, such as hydrogen bonding and van der Waals forces between PAM and CNF [39]. These observations provide evidence for the successful binding of CNF and PAM. The intensities of the characteristic peaks gradually increased with rising NaOH concentration, and the N-H bending vibration absorption peaks of PAM at 1647 cm^−1^ and 1597 cm^−1^ shifted to 1652 cm^−1^ and 1605 cm^−1^, respectively. This phenomenon can be attributed to the partial hydrolysis of the amide group (-CONH2) to the carboxylic acid groups (-COOH) in polyacrylamide following exposure to high concentrations of NaOH. Subsequently, these carboxylic acid groups reacted with NaOH to form sodium carboxylate (-COONa). It is well established that the introduction of carboxylic acid groups onto polymers results in a screening effect, thereby reducing both the solution viscosity and the mechanical strength of the CNF-PAM [40]. This is also evident from the appearance of the characteristic C=O stretching vibration of carboxylates at 1700 cm^−1^ on the FTIR spectrum when the NaOH concentration is increased to 10%.

Thermogravimetric (TG) and derivative thermogravimetric (DTG) analyses were conducted on both the CNF-PAM and the NaOH-treated CNF-PAM, with the results presented in Figure 3c and Figure 3d, respectively. From the TG curves, it is evident that the decomposition of the NaOH-treated samples occurs more prominently at lower temperatures (~220 °C) compared to PAM and the CNF-PAM. Additionally, the extent of decomposition increases with higher concentrations of NaOH. However, after 420 °C, the mass loss of the gel decreased with the increasing NaOH treatment concentration. From Figure 3c, it can be observed that the mass loss rates of PAM and the CNF-PAM are nearly identical, indicating that the addition of CNF does not significantly affect the thermal stability of PAM. Specifically, at 800 °C, the mass loss rate of the CNF-PAM is approximately 80%, while that of the CNF-PAM10% is 74%. These findings suggest that the NaOH-treated gels exhibit satisfactory thermal stability. Figure 3d illustrates a more complex pyrolysis profile, with a minor degradation peak occurring around 150 °C attributed to water loss, marking the initial stage of the degradation process. Subsequently, a degradation peak appeared at 196 °C, which was mainly due to the degradation of the glucose/glucuronide copolymer molecular chains in the amorphous region of the CNF and the degradation in the crystalline region [41,42], followed by the beginning of the decomposition of the main chains in the PAM. When treated with NaOH, the rate of degradation was significantly reduced and the peak shifted to 240 °C, indicating the onset of the second stage of degradation. The third stage primarily occurs within the temperature range of 272–327 °C, with pyrolysis peaks shifting towards higher temperatures. These changes in pyrolysis behaviour indicate that the chemical structure of the CNF-PAM gels following the NaOH treatment exhibits greater stability, thereby enhancing their thermal stability [43].

**Figure 4:**
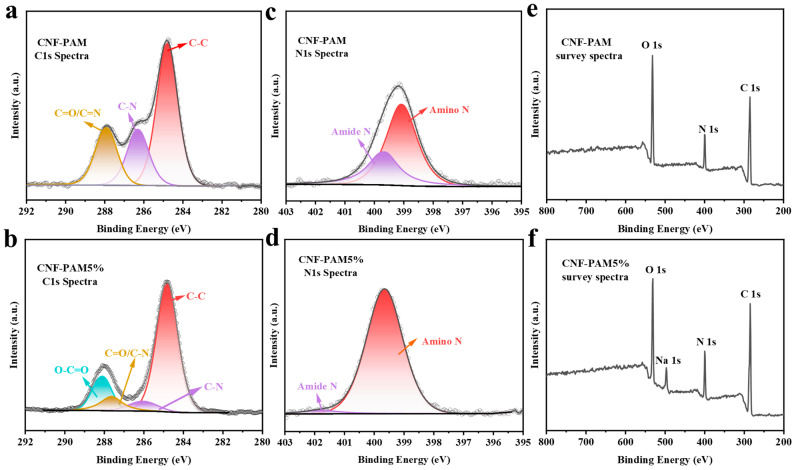
X-ray photoelectron spectroscopy (XPS) of the gels, including high-resolution C 1s spectra for the CNF-PAM and the CNF-PAM5% (**a**,**b**); high-resolution N 1s spectra (**c**,**d**); and XPS survey spectra (**e**,**f**).

In addition, XPS spectroscopic analyses were conducted on the CNF-PAM and the CNF-PAM5% to examine the chemical changes induced by the NaOH treatment. As illustrated in Figure 4a, the C 1s spectrum of the CNF-PAM comprises three distinct peaks: C–C (284.80 eV), C–N (286.30 eV), and C=O/C=N (287.92 eV). Compared to the CNF-PAM, the CNF-PAM5% (Figure 4b) exhibited an O–C=O peak at 288.10 eV, which can be attributed to the carboxylic acid groups formed through the hydrolysis of the CNF-PAM. The presence of two peaks, amino N (399.07 eV) and amide N (399.65 eV), on the surface of the CNF-PAM (Figure 4c), along with the overall leftward shift in binding energy after the NaOH treatment (Figure 4d), further confirms the hydrolysis of the CNF-PAM. Meanwhile, the presence of elemental Na on the surface of the CNF-PAM5%, as evidenced by a comparison of the survey spectra (Figure 4e,f), suggests that the CNF-PAM5% underwent hydrolysis and reacted with NaOH to form sodium carboxylate. However, no distinct characteristic peaks indicative of hydrolysis were observed in the FTIR, suggesting that the hydrolysis of the CNF-PAM at 5% concentration is minimal. This indicates that 5% NaOH concentration may be at the threshold for initiating the CNF-PAM hydrolysis.

### 2.2. Adsorption Kinetics Analysis

**Figure 5:**
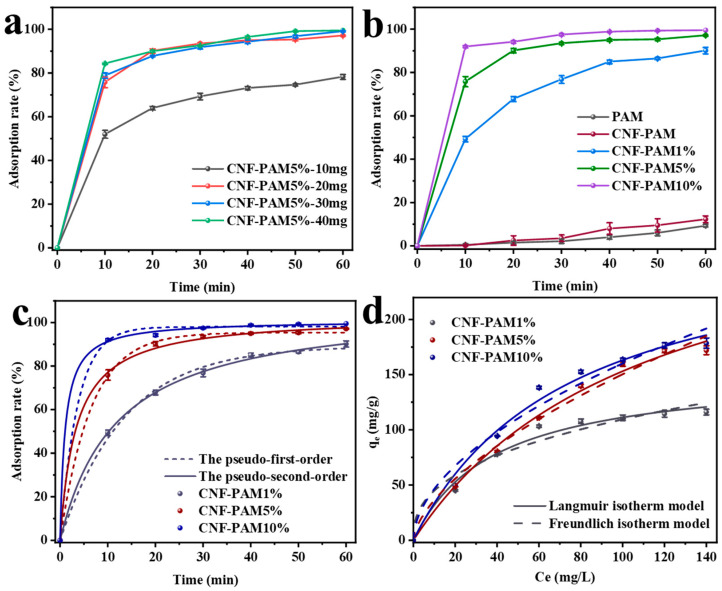
Adsorption rate of MB by the different PAM-CNF5% deliveries (**a**); variation in the adsorption rate of MB by the gel at different time (**b**); the pseudo-first-order and the pseudo-second-order kinetic models for MB adsorption by gels (**c**); adsorption data fitted to the Langmuir and Freundlich isotherm models (**d**).

The effects of varying the CNF-PAM5% delivery amounts (10 mg, 20 mg, 30 mg, 40 mg) on the MB adsorption rate are demonstrated in Figure 5a. It is evident that the adsorption rate of MB in the solution exhibited an increase with the increasing amount of the CNF-PAM5% placed in the solution, from 78.0% to 99.5%. Furthermore, the figure demonstrates a gradual stabilisation of MB adsorption in the solution by the CNF-PAM5% when the delivery amount exceeds 20 mg. Consequently, from the perspectives of economic feasibility and practical application, it is more appropriate to determine the dosage of the CNF-PAM5% as 20 mg (20 mg/L, 50 mL). The adsorption rate and capacity of the gel are critical factors that determine its practical value and applicability. Figure 5b illustrates the adsorption behaviour of different gels at various time points. The adsorption efficiency of PAM and the CNF-PAM was relatively low, with a maximum adsorption rate of only 12.3%. This can be attributed to the formation of a highly stable gel network between PAM and CNF, resulting in smaller pore sizes that prevented MB from penetrating into the interior of the aerogel and accessing more active sites. When treated with NaOH, the adsorption capacity of all samples increased rapidly within 10 min and showed a positive correlation with the NaOH concentration, reaching 92% at a sodium hydroxide concentration of 10%. This is attributed to the conversion of amide groups in PAM to carboxylate groups by the NaOH treatment, which enhances the negative charge on the gel surface. Simultaneously, we examined the surface charge of CNF [37] and discovered that the prepared CNF exhibited a negative charge. Therefore, in addition to chemical interactions, the increased exposure of CNF resulted in electrostatic attraction between MB and the gel, thereby enhancing its adsorption efficiency. Furthermore, gel swelling loosened the network structure and increased the porosity of the gel, thereby exposing more active sites. Figure 5c illustrates the application of pseudo-first-order and pseudo-second-order kinetic models to examine the influence of adsorption time on adsorption capacity (citation of kinetic equations [10]). The specific fitting coefficients R^2^ are presented in Table 1. Notably, the R^2^ value for the pseudo-second-order kinetic model is significantly closer to 1 compared to that of the pseudo-first-order model. This suggests that the adsorption of MB by the gel is predominantly chemisorption. The fitted isothermal adsorption curves provide insight into the distribution of MB molecules between the solid and liquid phases upon reaching adsorption equilibrium [44]. As illustrated in Figure 5d, the adsorption of MB by the gel was analysed using both Langmuir and Freundlich isotherm models. The Langmuir model suggests monolayer adsorption, in contrast to the Freundlich model, which implies heterogeneous surface adsorption [45]. Based on the fit results presented in Table 2, the R^2^ value of the Langmuir model is significantly closer to 1 compared to that of the Freundlich model. This suggests that the adsorption of MB on the gel surface is more likely to occur through monolayer adsorption. Once the adsorption sites on the gel surface are occupied by MB, no additional adsorption can occur at these active sites [46]. From the data, it was determined that the maximum adsorption capacity of the CNF-PAM10% is 178.50 mg/g. Additionally, the adsorption capacity of the gel increased as the NaOH concentration rose, suggesting a promising approach for developing simple and cost-effective gel adsorbents.

### 2.3. Effect of Different Factors on Adsorption Performance

**Figure 6:**
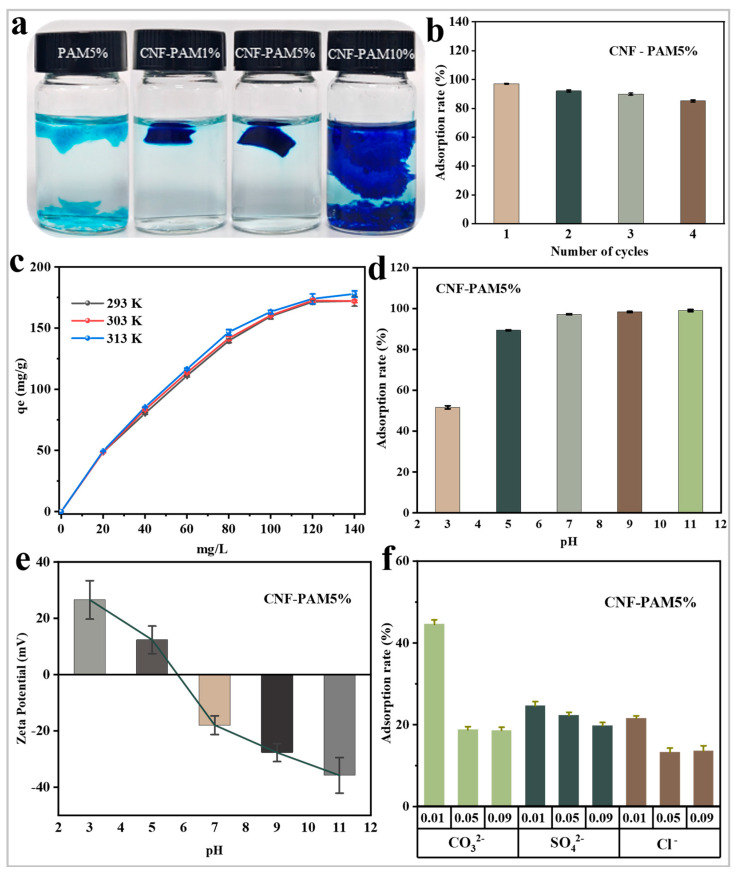
State of different gels after 60 min of adsorption (**a**); cyclic adsorption performance of MB by the CNF-PAM5% gel (**b**); influence of varying temperatures on the adsorption of MB by the CNF-PAM5% (**c**); impact of different pH levels on the adsorption of MB by CNF-PAM5% (**d**); zeta potential of the CNF-PAM5% at pH values ranging from 3 to 11 (**e**); effect of interfering anions on the adsorption of MB by the CNF-PAM5% (**f**).

The gel undergoes water shock and repeated swelling during the adsorption process. Therefore, it is crucial for the gel to maintain its integrity post-adsorption, which facilitates recovery and reuse. In the experiment, the gel was retrieved and photographed after 60 min of adsorption. As shown in Figure 6a, the PAM5% gel without added CNF adsorbed and completely fragmented after 60 min, and the gel remained monolithic when the NaOH treatment concentration was 1% and 5% after the addition of CNF. This indicates that the incorporation of CNF not only mitigated the hydrolysis of PAM but promoted the integration of dense cellulose filaments into the PAM gel network. As a result, the overall structural stability was significantly enhanced under conditions of agitation and water impact. These findings are in agreement with the results derived from the SEM analysis. To evaluate the recoverable performance of the gel, cyclic adsorption experiments were conducted on the CNF-PAM5% (Figure 6b). The results indicated that the adsorption efficiency remained at 85% after four cycles. Additionally, the CNF-PAM5% sample retained its integrity without any signs of fragmentation following 4 h of stirring and water impact. Therefore, even though the CNF-PAM10% had the highest adsorption capacity, the CNF-PAM5% had the best overall performance, as analysed by the adsorption performance test and cell cycling test. In addition, the influence of extrinsic factors on the adsorption process was examined by varying the temperature, pH, and introducing different impurity anions. As illustrated in Figure 6c, altering the temperature had a minimal impact on the gel adsorption of MB. As shown in Figure 6d, changing the pH had a large effect on the adsorption. The adsorption performance of the CNF-PAM5% was increasing at pH > 7 of the solution and vice versa was decreasing. To investigate the underlying reasons for this phenomenon, the zeta potential of the CNF-PAM5% was measured across a range of pH values. As demonstrated in Figure 6e, the surface charge of the CNF-PAM5% undergoes a shift from positive to negative as the pH increases from 3 to 11. This transformation is presumably attributable to the deprotonation of functional groups on the CNF-PAM5% surface in an alkaline environment, which results in an increase in negative charge density and, consequently, an enhancement in electrostatic attraction between the negatively charged gel surface and the positively charged MB molecules [47]. At low pH levels, besides the protonation of the gel surface, the abundant H⁺ ions in the solution may compete with MB⁺ for adsorption sites, thereby contributing to the reduced MB adsorption efficiency. The effect on the adsorption performance as the pH changes plays an important role in the cyclic (adsorption–desorption) use of samples. In addition to the aforementioned experiments, various impurity anions were introduced into the adsorption tests. As illustrated in Figure 6f, the adsorption capacity decreases gradually with the increasing concentration of interfering ions. This phenomenon can be attributed to two factors: firstly, the addition of ions alters the surface charge distribution of the gel, thereby reducing its affinity for MB. Secondly, the charged ions may occupy the adsorption sites on the gel surface, leading to a reduction in available binding sites and consequently decreasing the overall adsorption capacity.

### 2.4. Adsorption Mechanism Analysis

**Figure 7:**
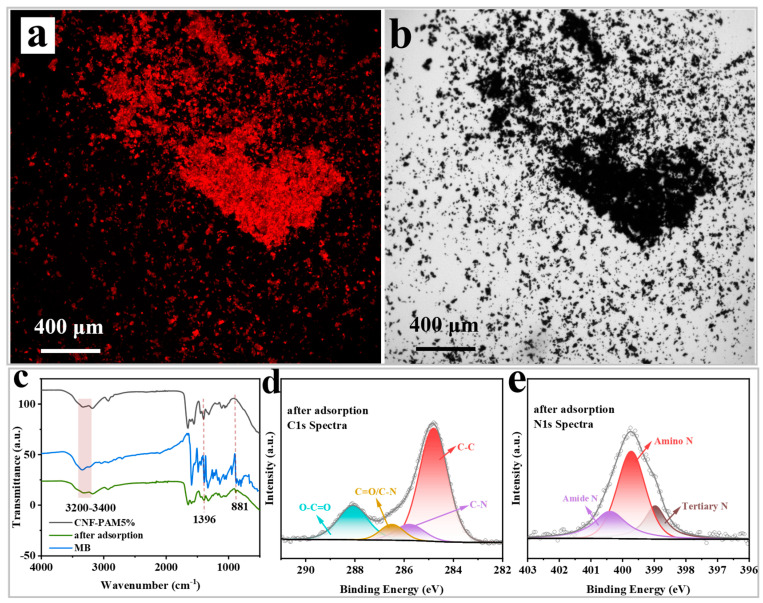
Comparison of CNF-PAM5% under fluorescence microscope after MB adsorption (**a**,**b**). FTIR spectra of CNF-PAM5% before and after MB adsorption (**c**); C 1s high-resolution XPS spectra (**d**); and N 1s high-resolution XPS spectra (**e**) after MB adsorption by CNF-PAM5%.

As MB is capable of emitting fluorescence when observed under the microscope, it can be seen in Figure 7a,b that the CNF-PAM5% has successfully adsorbed the MB dye. As illustrated in Figure 7c, the FTIR spectrum demonstrates that the absorption peaks located at 3320–3400 cm^−1^ and 1396 cm^−1^ experience a substantial decline in intensity following MB adsorption. Additionally, the C–H bond stretching vibration of MB manifests at 881 cm^−1^ in comparison with the gel prior to adsorption. The results obtained demonstrate that MB was successfully adsorbed on the surface of the CNF-PAM5% gel, and that the chemical reaction between the adsorption was the result of bond-to-bond interactions [48]. In addition to this, the XPS spectra demonstrated that the peaks of C–N and C=O/C–N of the C 1s spectrum following adsorption underwent a shift (Figure 7d), and a tertiary nitrogen peak emerged at 398.97 eV in the N spectrum after the adsorption of MB (Figure 7e), thereby reiterating the efficacy of MB adsorption on the surface of the CNF-PAM5%. In conclusion, the FTIR spectra, zeta potential, and XPS tests demonstrate that, following the NaOH treatment, there is an increase in the functional groups on the surface of the CNF-PAM, as well as a conversion of some of the amide groups in the PAM into carboxylate groups, resulting in an increase in the negative charge on the gel surface. Secondly, the prepared CNF also carries a large amount of negative charge, which enhances the electrostatic adsorption between the CNF-PAM5% and MB. Therefore, the adsorption of MB by the CNF-PAM5% was caused by both physical and chemical effects.

### 2.5. Toxicity Analysis of the Gels

**Figure 8:**
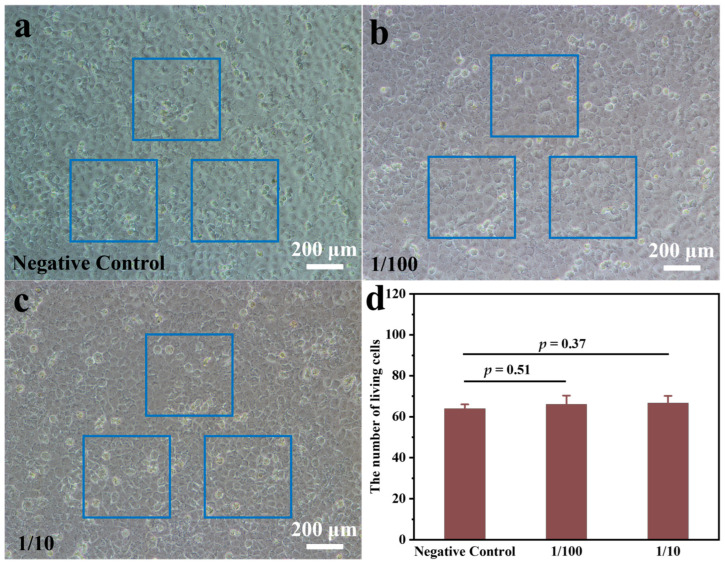
The HPDE cells were treated with a medium containing CNF-PAM5% and observed under the microscope. HPDE cells were cultured in a normal cell culture medium as a negative control for 48 h (**a**); HPDE cells were cultured in a cell culture medium containing 1/100 *v*/*v* CNF-PAM5% for 48 h (**b**); HPDE cells were cultured in a cell culture medium containing 1/10 *v*/*v* CNF-PAM5% for 48 h (**c**); Randomly select three areas of the same size from the microscope field of view as three repeated experiments, observe and count the number of live cells in (**a**–**c**). The calculation of the *p*-value is based on the Student’s *t*-test. bar = 200 μm (**d**).

The utilisation of gel adsorbents in the context of wastewater treatment represents a significant methodology. However, a concomitant consideration pertains to the potential toxicity of these materials, which can exert a deleterious effect on water bodies. In the event of the adsorbed completed gel being utilised as a fish fungicide to release MB, the biocompatibility index of the gel becomes of particular significance. As demonstrated in Figure 8, the gel was subjected to a cell viability assay. In this assay, the HPDE cell culture medium was combined with varying volumes of the CNF-PAM5%, and the results indicated that there was no substantial cell death after a 48-h incubation period. The toxicity of the CNF-PAM5% on the HPDE cells was assessed by quantifying the number of viable cells per unit area. The number of cells per unit area in the absence of the CNF-PAM5% was 64 viable cells (average of 3 viable cell numbers per unit area) (Figure 8a). With the incorporation of 1/100 volume of the CNF-PAM5%, the number of cells per unit area was approximately 66 live cells (Figure 8b). Following an increase in the volume ratio of the CNF-PAM5% to 1/10, the number of cells per unit area was 66.7 live cells (Figure 8c). The results demonstrated that there was no significant difference (*p* > 0.05) in the number of viable cells after the addition of 1/10 or 1/100 of the CNF-PAM5% (Figure 8d), thus substantiating the hypothesis that the CNF-PAM5% exhibits excellent cytocompatibility for HPDE cells.

### 2.6. Prospects for the Application of Gel-Releasing MB as a Fish Fungicide

**Figure 9:**
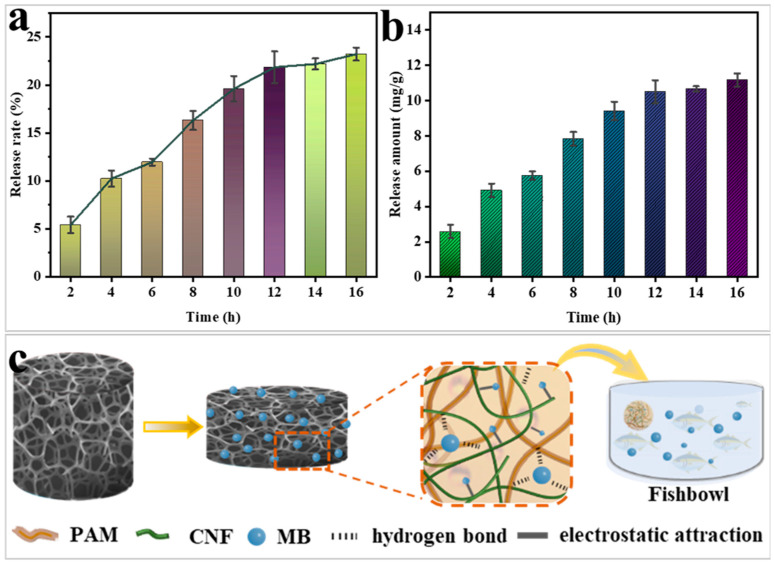
MB release rate of CNF-PAM5% over time (**a**); the release of MB over time (**b**). Mechanistic diagram of applying the gel to fish sterilisation (**c**).

As demonstrated in Figure 9a,b, the gel exhibits a gradual release of MB following the completion of adsorption. As the release time reached 12 h, the release rate of MB was found to be 21.9%, with a release amount of 10.5 mg/g. There was no significant increase in the release rate in the following time. The aforementioned phenomenon can be attributed to two factors. Firstly, the aqueous solution of MB becomes alkaline. Secondly, when the adsorbed gel is immersed in deionised water, the difference in solution concentration results in the dislodgement of the poorly adsorbed MB. The weak change in pH also affects the surface charge of the gel, leading to the detachment of MB. This is the reason why the release almost stops after 16 h. The specific adsorption-release mechanism is illustrated in Figure 9c. It is well known that MB is an effective fish fungicide, but there are strict requirements on the dosage of MB. In this study, the gel was first repeatedly adsorbed with MB to maximise its utilisation efficiency and then released into the fish tank as a fish fungicide, and the slow-release process not only controlled the dosage of MB but made it easier to observe the state of the fish as the MB dosage was increased. This experimental release utilised a solution with a concentration of 20 mg/L and a volume of 50 mL. The gel adsorbent has the advantage that the concentration of the solution can also be altered to suit the desired biocide delivery. This results in an additional application of merit to the gel adsorbent.

## 3. Conclusions

In conclusion, the interpenetrating network gel prepared from CNF and PAM was modified using NaOH to obtain a low-toxicity, simple to synthesise, inexpensive, recoverable, and recyclable adsorbent for dye wastewater treatment. In this study, CNF was incorporated into the PAM system, resulting in the interpenetration of the fibre filaments of CNF and PAM to form a network structure with favourable mechanical properties. The gel was then modified using NaOH to improve the functional groups and charges on the surface of the gel, thus giving it good adsorption-release properties for MB treatment. Furthermore, the gel exhibits favourable adsorption-release properties, with a maximum adsorption capacity of 172.08 mg/g for the CNF-PAM5%, in addition to demonstrating effective recyclability during the recycling process. In the toxicity analysis of the gel, it was demonstrated that, even when the sample volume of the CNF-PAM5% was increased up to 10 times, there was no significant difference in the amount of cellular activity, and the gel did not increase the toxicity burden during use. Consequently, the gel adsorbent material prepared in this study is anticipated to be utilised in the domain of fish biocides following the completion of adsorption in the field of dye effluents. The controllable time and dosage delivery of this gel material may facilitate the observation of the health and survival of fish.

## 4. Materials and Methods

### 4.1. Materials

In our previous research, we successfully prepared a cellulose nanofibril (CNF) with a solid concentration of 8%, which was used in this study. The details of the preparation method and characterisation are provided in Reference [33]. Polyacrylamide (PAM) was purchased from Fuchen Chemical Reagent Co., Ltd. (Tianjin, China), whilst N, N-methylene acrylamide (MBA), and ammonium persulfate (APS) were procured from Shanghai Aladdin Biochemical Technology Co., Ltd. (Shanghai, China), and sodium hydroxide (NaOH) was obtained from Tianli Chemical Reagent Co., Ltd. (Tianjin, China), while methylene blue (MB) and hydrochloric acid (HCl) were obtained from Guangfu Chemical Reagent Co., Ltd. (Tianjin, China). All chemicals utilised in this study were of analytical grade, thus obviating the necessity for further purification. Deionised water was used throughout this study.

### 4.2. Preparation Methods

AM (2.7 g), APS (0.0224 g), and MBA (0.01 g) were added to 5 mL of deionised water and stirred for 2 h. Then, 10 mL of CNF dispersion was slowly added and stirred for 6 h. The solution obtained was poured into the mould and placed in a sealed package in a constant temperature and humidity chamber at 60 °C for 6 h. The gel obtained above was cut into slices of 1 cm diameter. The gel discs were then placed in 1%, 5%, and 10% NaOH solutions and left to stand for 2 h. The finished soaked gel discs were then removed and washed repeatedly with deionised water and immersed in deionised water until the pH reached 7. The samples were designated as CNF-PAM1%, CNF-PAM5%, and CNF-PAM10%, respectively. Meanwhile, the samples devoid of CNF were designated as PAM, and the samples that underwent no NaOH treatment were designated as CNF-PAM.

### 4.3. Characterisation and Testing of Gels

The morphology of the nanocellulose (CNF) treated with different NaOH concentrations (1%, 5%, 10%) was characterised using transmission electron microscopy (TEM, JEM-2100, JEOL Ltd., Kyoto, Japan). The prepared samples were then freeze-dried using a freeze-dryer (Shuangjia Instrument Co., Ltd., Ningbo, China) and subsequently used. The samples were then subjected to microscopic analysis using a scanning electron microscope (SEM, Apreo S HiVac, Thermo Fisher Scientific, Waltham, MA, USA). The samples were tested using an X-ray diffractometer (XRD, XRD-6100, Shimadzu Corporation, Kyoto, Japan). The gels were analysed using Fourier infrared spectroscopy (FTIR, Spectrum 400, Perkin Elmer, Waltham, MA, USA). The thermal stability was evaluated using simultaneous thermal infrared analysis (STA 6000-SQ8, Perkin Elmer, Waltham, MA, USA). The tensile properties of the hydrogels were examined using a universal mechanical testing machine (CMT 6104, Sansi Eternal Technology Co., Ltd., Ningbo, China). The pH of the solution to be tested was then adjusted with 0.1 M HCl and 0.1 M NaOH, after which the potential changes were analysed using a nanoparticle size potentiometer (Nanotrac wave II, Microtrac, Microtrac Inc., Largo, FL, USA). The adsorption-release of MB on the gel was evaluated using a UV-visible spectrophotometer (UV-vis, TU-1950, Beijing Persee Instrument Co., Ltd., Beijing, China). Photographs of the changes before and after gel adsorption were taken using a high-resolution cell imager (excitation wavelength: 665 nm, Thunder, Leica, Wetzlar, Germany). The valence and chemical composition of each element was analysed using an X-ray photoelectron spectrometer (XPS, Thermo Scientific K-Alpha, Thermo Fisher Scientific, Waltham, MA, USA).

### 4.4. Adsorption Rate and Adsorption Capacity Tests

The adsorption rate and the amount of adsorption of the gel were investigated by controlling the mass of the gel, the concentration of the initial MB, the time of adsorption, different MB concentrations, different pH values, temperature, and ions. Firstly, lyophilised gel PAM-CNF 5% (0.01 g, 0.02 g, 0.03 g, 0.04 g) was added to MB solution at a concentration of 20 mg/L, 50 mL for the adsorption test. The optimal delivery amount for adsorption was explored by observing the change in the adsorption rate over time. Next, the adsorption test was conducted by incorporating freeze-dried gel (0.02 g) into a solution of MB (50 mL) at a concentration of 20 mg/L. The rate of adsorption was evaluated for various gels over time. Adsorption experiments were conducted on MB solutions of varying concentrations (20 mg/L, 40 mg/L, 60 mg/L, 80 mg/L, 100 mg/L, 120 mg/L, 140 mg/L) using a selection of gels, with the aim of determining the maximum adsorption capacity. In the subsequent experiment, the pH (3.0–11.0) of the 20 mg/L MB (50 mL) solution was adjusted using 0.1 M HCl and 0.1 M NaOH, with the objective of determining its effect on the adsorption. Subsequently, varying the concentrations of NaCl, Na_2_SO_4_, and Na_2_CO_3_ (0.01 M, 0.05 M, and 0.09 M) were introduced to the MB solution until the adsorption reached equilibrium. Thereafter, the impact of ionic strength on the adsorption process was investigated. Adsorption thermodynamic experiments were conducted at temperatures of 293 K, 303 K, and 313 K. Subsequently, a cyclic loading test was performed on the gel (20 mg/L MB, 50 mL) solution, which was washed by repeated immersion in a weakly acidic solution and deionised water during unloading. The concentration of MB was determined spectrophotometrically at a wavelength of 665 nm. The specific adsorption capacity was calculated using the following Equations (1) and (2):

(1) $$Adsorption\;rate=\frac{{(C}_{0}-C_{e})}{C_{0}}\times 100\%$$

(2) $$q_{e}=\frac{{(C}_{0}-C_{e})\times V}{m}$$

where *q_e_* (mg/g) denotes the adsorption capacity of the gel at the equilibrium moment, *C*_0_, *C_e_* (mg L^−1^) are the initial MB concentration and the concentration at the equilibrium moment, respectively, *V* (L) is the volume of the initial MB solution, and *m* (g) is the mass of the gel.

### 4.5. Release Rate Test

Three sets of 0.02 g of the CNF-PAM5% gels were each placed into 50 mL of the MB solution (concentration of 20 mg/L), and the samples were taken out to test their concentration after 60 min of adsorption. Then the adsorbed gels were put into 50 mL of deionised water for release experiments, respectively. The release rate was calculated by Formulas (3) and (4):

(3) $$Release\;rate=\frac{C_{t}}{20-C_{1}}\times 100\%$$

(4) $$Release\;amount=\frac{C_{t}\times V}{m}$$

where *C_t_* denotes the concentration at the moment of release *t*, *C*_1_ denotes the concentration after 60 min of gel adsorption, and *m* (g) is the mass of the gel.

### 4.6. Kinetic Modelling and Isothermal Curve Fitting

The effect of the adsorption time on the adsorption capacity was investigated using pseudo-first-order (Equation (5)) and pseudo-second-order (Equation (6)) kinetic model fits [10].

The pseudo-first-order kinetic model is as follows:

(5) $$q_{t}=q_{e}\left(1-e^{-k_{1}t}\right)$$

The pseudo-second-order kinetic model is as follows:

(6) $$q_{t}=\frac{q_{e}^{2}k_{2}t}{1+q_{e}k_{2}t}$$

where *k*_1_ (h^−1^) and *k*_2_ (g mg^−1^ h^−1^) are the reaction rate constants for pseudo-first-order and pseudo-second-order kinetic models, respectively.

The adsorption equilibrium data were fitted using the Langmuir (Equation (7)) and Freundlich (Equation (8)) isothermal models to analyse the adsorption of the gel on MB [10].

The Langmuir model is as follows:

(7) $$q_{e}=\frac{q_{max}k_{L}C_{e}}{1+k_{L}C_{e}}$$

The Freundlich model is as follows:

(8) $$q_{e}=k_{F}C_{e}^{1/n}$$

where *q_e_* and *q_max_* (mg g^−1^) are the adsorbed amount of the sample at adsorption equilibrium and the maximum adsorption capacity of the sample, respectively, *C_e_* (mg L^−1^) is the concentration at adsorption equilibrium, *k_L_* and *k_F_* are the Langmuir and Freundlich model constants, respectively, and *1*/*n* represents the empirical constant.

### 4.7. Toxicity Test of the Gel on Living Cells

The experimental methodology was founded upon the international standard ISO 10993-5 [49], and employs biological parameters to evaluate the in vitro biological response of mammalian cells. The CNF-PAM5% was utilised for the test samples, and the specific experimental methodology was delineated in reference [37,50,51]. The CNF-PAM5% samples were crushed and ground into 24-well plates. A high sugar complete culture medium (DMEM, SH30022.01B, HyClone, Logan, UT, USA) containing 10% foetal bovine serum (FBS, CTCC-002-020, Meisen CTCC, Hangzhou, China), 1% glutamine (Gibco, Thermo Fisher Scientific, Waltham, MA, USA), and 1% penicillin and streptomycin (SV30010, HyClone, Logan, UT, USA) was added to each well, and the final volume was filled with high sugar medium DMEM. An amount 0.5 mL of HPDE (human pancreatic epithelial cells, Shanghai Hongshun Biotechnology Co., Ltd., Shanghai, China) cells were inoculated into 24-well plates, maintaining a consistent concentration of added cells. By controlling the volume ratio of the CNF-PAM5% to other media and other components, the cells were co-cultured at 37 °C for 48 h and observed under the microscope. The number of live cells in the same area was counted in the wells with different volume ratios. The experiments without the addition of the CNF-PAM5% samples were used as negative controls.

## Figures and Tables

**Table 1:** Parameters related to adsorption kinetics.

Pseudo-First-Order					Pseudo-Second-Order		
	q_e,exp_ (mg·g^−1^)	q_e,c_ (mg·g^−1^)	k_1_ (h^−1^)	R^2^	q_e,c_ (mg·g^−1^)	k_2_ × 10^4^ (g·mg^−1^·h^−1^)	R^2^
CNF-PAM1%	45.08	89.14192	0.07433	0.99607	108.17313	0.000776841	0.99925
CNF-PAM5%	48.58	95.40894	0.15557	0.99874	103.07561	0.00287	0.99911
CNF-PAM10%	49.75	98.04909	0.27116	0.99752	101.02541	0.00928	0.99941

**Table 2:** Adsorption isotherm parameter.

Langmuir Isotherm				Freundlich Isotherm		
	q_m_ (mg g^−1^)	k_L_ (L mg^−1^)	R^2^	n	k_F_ (L mg^−1^)	R^2^
CNF-PAM1%	116.08	0.02614	0.95173	2.58906	18.47327	0.94484
CNF-PAM5%	172.08	0.00892	0.98491	1.62024	8.76337	0.98246
CNF-PAM10%	178.50	0.0133	0.96972	1.86043	13.47115	0.96474

## Data Availability

The data presented in this study are available on request from the corresponding author or co-authors. The data are not publicly available.
